# Molecular and Epidemiological Surveillance of *Sporothrix* spp. in Cats and Dogs from Southern Brazil

**DOI:** 10.1007/s11046-026-01107-z

**Published:** 2026-09-22

**Authors:** Gabriela Ladeira Sanzo, Renata Osório de Faria, Juliana Tasende Ferrando, Marcela Brandão Costa, Isabela de Souza Morales, Giulia Batista Freitas, Lara Costa Grumann Michel, Luana Pereira Ramirez, Sergio Jorge, Kauê Rodriguez Martins, Rodrigo Casquero Cunha, Clairton Marcolongo Pereira, Angelita dos Reis Gomes

**Affiliations:** 1 Faculty of Veterinary Medicine, Federal University of Pelotas, Pelotas, Rio Grande Do Sul Brazil; 2 Department of Preventive Veterinary Medicine, Faculty of Veterinary Medicine, Federal University of Pelotas, Pelotas, Rio Grande Do Sul Brazil; 3 Department of Veterinary Clinics, Faculty of Veterinary Medicine, Federal University of Pelotas, Pelotas, Rio Grande Do Sul Brazil; 4 Veterinary Pathology Laboratory UNESC, Faculty of Veterinary Medicine, University Center of Espírito Santo (UNESC), Colatina, Espírito Santo Brazil; 5 Faculty of Veterinary Medicine, Center for Diagnosis and Research in Veterinary Mycology, Federal University of Pelotas, Pelotas, Rio Grande Do Sul Brazil

**Keywords:** Sporotrichosis, *Sporothrix brasiliensis*, Cross-border zoonoses, Molecular epidemiology, Rio Grande do Sul, Public health surveillance

## Abstract

*Sporotrichosis*, a subcutaneous mycosis caused by *Sporothrix* spp., has emerged as a major zoonotic disease in Brazil, primarily driven by *Sporothrix brasiliensis*. The southern state of Rio Grande do Sul exhibits distinctive ecological and climatic conditions that may shape the persistence and distribution of this pathogen in this region. This study conducted molecular and epidemiological surveillance of feline and canine sporotrichosis in southern Rio Grande do Sul, Brazil. A total of 100 isolates from the culture collection of the Veterinary Mycology Laboratory at the Federal University of Pelotas (UFPel) were analyzed by PCR targeting the *calmodulin* (CAL) gene for species identification. Fourteen representative isolates were sequenced and subjected to phylogenetic analysis using the Maximum Likelihood method, including reference sequences from Brazil, Paraguay, and Argentina. Epidemiological and clinical variables, including host species, sex, lesion site, and morphology, were evaluated to characterize the epidemiological and clinical profiles of sporotrichosis in the study region. *S. brasiliensis* and *S. schenckii* were identified in 97% and 3% of the isolates, respectively. The CAL-based phylogeny confirmed the monophyly of *S. brasiliensis* and revealed low intraspecific variability with regional clustering among southern Brazilian isolates. When interpreted within the Southern Cone context, the topology indicated a close genetic affinity between isolates from Rio Grande do Sul and reference sequences from Argentina and Paraguay. These findings confirm the territorial expansion of *S. brasiliensis* across southern Brazil and emphasize the importance of molecular surveillance for detecting and tracking the circulating lineages. Continuous regional monitoring is crucial for strengthening *One Health* strategies and mitigating the zoonotic spread of sporotrichosis in the Southern Cone.

## Introduction

Sporotrichosis, a subcutaneous mycosis of significant and growing public health concern in Brazil, is caused by fungi of the genus *Sporothrix*. Over the past two decades, Brazil has become the epicenter of zoonotic sporotrichosis, largely because of the emergence of *Sporothrix brasiliensis*, a species recognized for its high virulence and zoonotic potential [1]. This pathogen has established a robust transmission cycle through domestic cats, which act as both reservoirs and vectors of infection, particularly in urban areas, where close human-cat interactions facilitate its spread [2].

In Rio de Janeiro, sporotrichosis has reached epidemic proportions, driven by frequent contact between humans and cats in densely populated areas with large feline populations [3]. São Paulo, another major hotspot, has experienced similar patterns, with studies indicating significant impacts on animal and human health due to proximity to infected cats [4]. In Espírito Santo, a coastal state in southeastern Brazil, increasing sporotrichosis cases have been linked to unregulated urban expansion and the rapid growth of feline populations [5].

Rio Grande do Sul (RS) is the primary epicenter of sporotrichosis in Brazil, alongside Rio de Janeiro, where the disease has a significant zoonotic impact [6, 7]. Situated in a temperate zone with unique ecosystems, such as the Pampa biome [8], the state has a high incidence of sporotrichosis, particularly among domestic cats [6]. Sporotrichosis has shown a marked increase in recent years, particularly in the southern and metropolitan regions of RS [9]. In RS, the disease affects both humans and animals, with cats being the primary source of zoonosis [10, 11].

Owing to a lack of mandatory reporting, the true prevalence remains unclear, although the disease is considered endemic, with cases reported in 40 cities across seven health districts [9]. The incidence of feline cases in Rio Grande increased significantly from 0.75 new cases per month in 2010 to 3.33 cases per month in 2014, highlighting the rapid urban spread [12]. Sporotrichosis in southern Brazil, particularly in RS, is an emerging public health issue with an increasing incidence and wide geographic distribution. Although specialized reference services have improved diagnosis and treatment [7], aggressive public health policies and greater awareness are essential to curb the spread of this zoonotic disease [1, 9].

However, studies specific to Rio Grande do Sul remain scarce, mainly limited to clinical case reports and general epidemiological data, and lack comprehensive molecular investigations of the diversity of *Sporothrix* species circulating in the region [13]. This gap in region-specific data hinders the identification of *Sporothrix* variants endemic to Southern Brazil and constrains the development of targeted public health interventions. Additionally, the geographical proximity of Rio Grande do Sul to Uruguay and Argentina, which also reported sporotrichosis cases [14–17], underscores the need for rigorous molecular surveillance and integrated public health strategies to mitigate cross-border transmission risks and strengthen regional health responses to sporotrichosis.

This study addresses critical gaps in the molecular epidemiology of *Sporothrix* spp. in southern Rio Grande do Sul, a region of strategic importance for monitoring and controlling sporotrichosis in Brazil. Situated at the border with Uruguay and Argentina, this area plays a pivotal role in cross-border transmission dynamics, positioning it as a focal point for surveillance of emerging and re-emerging zoonoses. By conducting a comprehensive analysis of species distribution, transmission dynamics, and clinical manifestations, this study situates regional epidemiological and clinical profiles within both national and international contexts. The findings presented herein provide a foundation for developing targeted control strategies and enhancing zoonotic risk management in ongoing epidemic scenarios.

## Methodology

The study was conducted in southern Rio Grande do Sul, Brazil, a temperate region (Köppen Cfa) bordered by Uruguay, Argentina, Santa Catarina, and the Atlantic Ocean. The area encompasses coastal plains, low hills, and the Lagoa dos Patos Basin, with mild winters, hot summers, and evenly distributed rainfall. The Pampa biome predominates, supporting extensive cattle ranching and agriculture that sustains sparsely populated cities such as Pelotas, Rio Grande, and Bagé. The region’s proximity to Uruguay and Argentina facilitates cross-border interactions and biodiversity exchanges through wetlands, such as the Banhado do Taim [8].

Clinical and epidemiological data were retrospectively retrieved from passive case submissions to the Veterinary Mycology Laboratory of the Federal University of Pelotas (MICVET/UFPel), a regional diagnostic reference center for animal sporotrichosis. The records corresponded to confirmed cases diagnosed between 2019 and 2023 and included host species (dog or cat), age, sex, municipality of origin, lesion type, affected anatomical site, and the presence or absence of respiratory signs. Lesions were classified according to two main criteria: (i) anatomical distribution, defined as localized (single site) or disseminated (multiple regions), and (ii) morphological pattern, categorized as ulcerative, nodular, mixed, or other presentations (e.g., abscesses and pustules). Each animal was assigned a predominant lesion category to avoid duplication and ensure consistency in the analysis.

### Fungal Isolation and Culture Conditions

A total of 100 *Sporothrix* isolates previously identified at the genus level were selected from feline and canine cases recorded between 2019 and 2023 in the Clinical Mycological Collection at the Mycology Laboratory of the Federal University of Pelotas (UFPel), Pelotas, Brazil. Primary identification was based on the macromorphological and micromorphological characteristics of *Sporothrix* spp. To confirm viability before molecular analysis, cryopreserved isolates were thawed and subcultured on Sabouraud Dextrose Agar (SDA; Becton, Dickinson and Company, Franklin Lakes, NJ, USA) supplemented with chloramphenicol (Sigma-Aldrich, St. Louis, MO, USA) and incubated at 25 °C for 7–14 days to allow the development of young, viable colonies suitable for subsequent DNA extraction.

### Molecular Identification and Characterization

Genomic DNA was extracted from colonies grown on Potato Dextrose Agar using Brazol reagent (LCG Biotecnologia, Brazil) according to the manufacturer’s instructions. Species identification was performed by PCR amplification of the *calmodulin* (CAL) gene using species-specific primers that generated a 469 bp fragment [18]. Each 20 µL reaction contained 2× PCR MasterMix (LCG Biotecnologia, Brazil), primers (3–10 pmol), template DNA, and nuclease-free water (NFW). Standard PCR cycling conditions were applied, and the amplicons were visualized on 1.5% agarose gels under UV illumination, and a 100 bp molecular weight marker (Ladder 100 bp 500 µl, Ludwig Biotechnology, Porto Alegre, Rio Grande do Sul, Brazil) was used. The PCR products were excised from the gel and purified using the Gel Purification Kit (Ludwig Biotechnology, Porto Alegre, Rio Grande do Sul, Brazil), following the supplier’s instructions. Sequencing was performed using the BigDye Terminator Cycle Sequencing Kit v3.1 (Thermo Fisher Scientific, USA) on an ABI 3500 Genetic Analyzer (Applied Biosystems, USA).

### Phylogenetic Analysis

The electropherograms were bidirectionally analyzed using the Phred base calling and Phrap assembly tool to obtain consensus sequences, which were subsequently aligned with the MEGA12 software (Molecular Evolutionary Genetics Analysis version 12) [19] using the ClustalW method. Sequence similarity searches were performed in the National Center for Biotechnology Information (NCBI) database using the BLAST tool (//blast.ncbi.nlm.nih.gov/Blast.cgi). The sequences obtained for the CAL gene were deposited in the NCBI GenBank under accession numbers PQ512086–PQ512099.

Phylogenetic relationships among *Sporothrix* isolates were inferred using the Maximum Likelihood (ML) method with the Tamura 3-parameter (T92) model [20], implemented in MEGA12 [21]. Rate heterogeneity among sites was modeled using a discrete gamma distribution, and *Sporothrix albicans* and *Ophiostoma rufum* were used as outgroups. Branch support was evaluated using 1000 bootstrap replicates [22], with values ≥ 60% considered significant. The final alignment included 27 sequences and 994 nt. Reference sequences from human and animal isolates from Brazil, Argentina, and Paraguay were retrieved from the NCBI GenBank database and included in the phylogenetic analysis for comparative purposes. The phylogenetic tree based on partial *CAL* sequences, a validated marker for discriminating species within the *Sporothrix schenckii* complex [18], showed *that S. brasiliensis* formed a well-supported monophyletic clade, clearly distinct from *S. schenckii* and *S. albicans*.

### Descriptive and Statistical Analysis

Descriptive statistics, including frequencies and percentages, were used to summarize categorical variables such as species distribution, sex, lesion morphology, anatomical site, and respiratory signs. Statistical analyses were performed using IBM SPSS Statistics version 25 (IBM Corp., Armonk, NY, USA). Associations between categorical variables were evaluated using the chi-square or Fisher’s exact test when the expected cell counts were < 5. Temporal trends in case numbers were assessed using the chi-square test. Comparative analyses between cats and dogs focused on lesion morphology and anatomical distribution, with statistical significance set at *p* < 0.05.

#### Ethical Approval

This study relied exclusively on archived *Sporothrix* isolates obtained from routine veterinary diagnostic submissions to the Veterinary Mycology Laboratory (MICVET/UFPel). No live animals or human subjects were directly involved, and no experimental procedures were performed for research. Therefore, approval by an Institutional Animal Care and Use Committee (IACUC) or an Institutional Review Board (IRB) was not required. All procedures complied with Brazilian regulations for clinical veterinary practice and the institutional biosafety standards of the Federal University of Pelotas (UFPel).

## Results

### Fungal Isolation and Case Composition

A total of 100 *Sporothrix* isolates were recovered from confirmed cases of feline and canine sporotrichosis diagnosed between 2019 and 2023 in southern Rio Grande do Sul, Brazil. All isolates originated from the clinical collection of the UFPel Veterinary Mycology Laboratory. Of these, 75 (75%) were from felines, 24 (24%) from canines, and one lacked species identification. Following revival from cryostorage, the colonies exhibited typical *Sporothrix* macromorphology after 7–14 days of incubation at 25 °C on Sabouraud Dextrose Agar, confirming viability for downstream molecular and phylogenetic analyses.

### Clinical and Epidemiological Findings

#### Temporal and Spatial Distribution

**Fig. 1:**
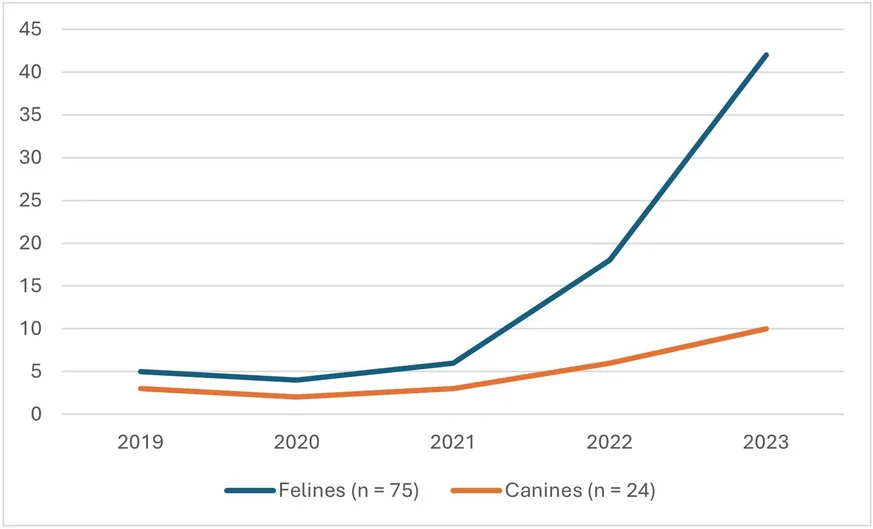
Temporal trends of feline (n = 75) and canine (n = 24) sporotrichosis cases diagnosed in southern Brazil between 2019 and 2023. A significant upward trend was observed for both hosts (χ^2^ for trend *p* < 0.001), with a sharper increase in feline cases after 2021, suggesting an expanding zoonotic transmission dynamic

Between 2019 and 2023, 99 sporotrichosis cases were diagnosed at the Veterinary Mycology Laboratory (MICVET/UFPel), including 75 feline and 24 canine cases. The number of confirmed cases progressively increased throughout the study period (Fig. 1). In felines, the number of cases increased from five in 2019 to 42 in 2023, representing an eight-fold increase. Canine cases also showed a steady upward trend, increasing from three in 2019 to ten in 2023. The temporal trend was statistically significant for both hosts (χ^2^ test for trend, *p* < 0.001), indicating sustained growth in the number of diagnosed sporotrichosis cases in the region. This pattern suggests an expanding local endemic scenario, with a marked increase in laboratory-confirmed cases after 2021. Information on housing conditions was available for 78 animals, including all 24 dogs. Among these, 73 (93%) were semi-domiciled, with regular outdoor access or free-roaming behavior. This profile reflects the typical management conditions observed in endemic urban areas and likely contributes to sustained exposure to infected animals and local transmission networks of *Sporothrix* spp.

**Fig. 2:**
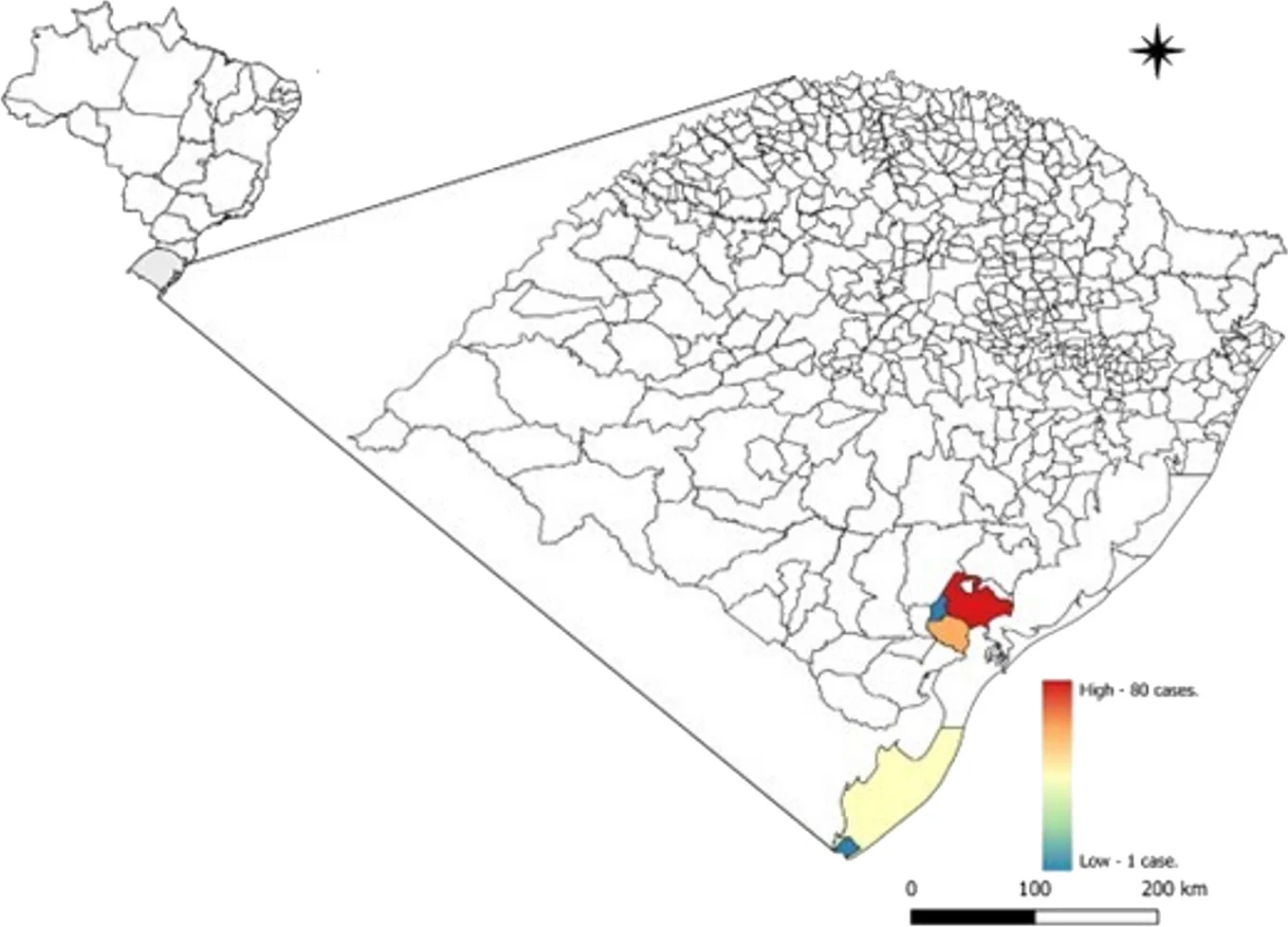
Geographic origins of the 100 *Sporothrix* spp. isolates analyzed from the culture collection of the Veterinary Mycology Laboratory (MICVET/UFPel), Pelotas, Southern Brazil (2019–2023). Warmer colors indicate municipalities that contributed more isolates, mainly Pelotas and its neighboring areas. The inset shows Rio Grande do Sul in Brazil, near Uruguay and Argentina. Data were derived from passive laboratory submissions and did not represent statewide surveillance

Therefore, the spatial distribution reflects the geographic origin of the submitted cases rather than an active surveillance design. Most cases originated from Pelotas and the surrounding municipalities, with sporadic submissions from other localities across the southern coastal region. Spatially, cases were highly concentrated in Pelotas (80%), followed by Capão do Leão (13%), with sporadic reports in Rio Grande, Morro Redondo, Santa Vitória do Palmar, and Chuí (1–3%). The spatial clustering of feline cases within the urban perimeter of Pelotas was statistically significant compared to the distribution of cases in all other municipalities combined (χ^2^(1) = 29.03, *p* < 0.0001) (Fig. 2).

#### Host, Sex, and Lesion Characteristics

The sex of all 99 animals was determined. Male cats were significantly more affected (52/75, 69.3%) than female cats (23/75, 30.7%) (χ^2^ = 12.13, *p* < 0.001). In dogs, infection showed no sex bias (16/24, 66.7% males vs. 8/24, 33.3% females, *p* = 0.102). Anatomical data were available for 71 cats (94.7%) and 24 dogs. In cats, disseminated lesions predominated (49.3%), followed by those on the muzzle (19.7%), face (11.3%), limbs (9.9%), and ears/neck (7.0%); other localized sites accounted for 2.8% of the cases. In dogs, disseminated and facial lesions were equally common (25.0% each), whereas muzzle (16.7%) and limb (12.5%) involvement was less frequent. Dissemination was significantly more frequent in cats (χ^2^ = 4.6p, *p*= 0.032) than in dogs. Lesion morphology also differed among hosts. In cats, ulcerative lesions predominated (53.0%), followed by nodular (22.7%), mixed (15.2%), and atypical (9.1%) lesions. In dogs, nodular lesions were the most frequent (41.7%), followed by ulcerative (33.3%), mixed (16.7%), and atypical (8.3%) forms, with significant differences between hosts (χ^2^ = 5.12, *p* = 0.024). Respiratory signs were observed in 9.3% of cats and 12.5% of dogs, with no interspecies difference (*p* = 0.589). Among cats with nasal lesions, 50% (7/14) presented with respiratory involvement. However, this association was not statistically significant (Fisher’s exact test, *p* = 0.754).

#### Molecular Identification of *Sporothrix* Species

PCR amplification of the *calmodulin* (CAL) gene produced the expected 469 bp amplicons in all isolates. The negative controls showed no amplification, confirming procedural sterility. Molecular typing identified *Sporothrix brasiliensis* as the predominant species (97/100; 97%), including 73 feline (97.3%) and 23 canine (95.8%) isolates from the total. *S. schenckii *sensu stricto was detected in three isolates (3%). No statistical association was observed between host species and identity (Fisher’s exact test, *p*=0.611). These findings corroborate *S. brasiliensis* as the dominant etiological agent of sporotrichosis in animals in southern Brazil.

#### Phylogenetic Analysis

**Fig. 3:**
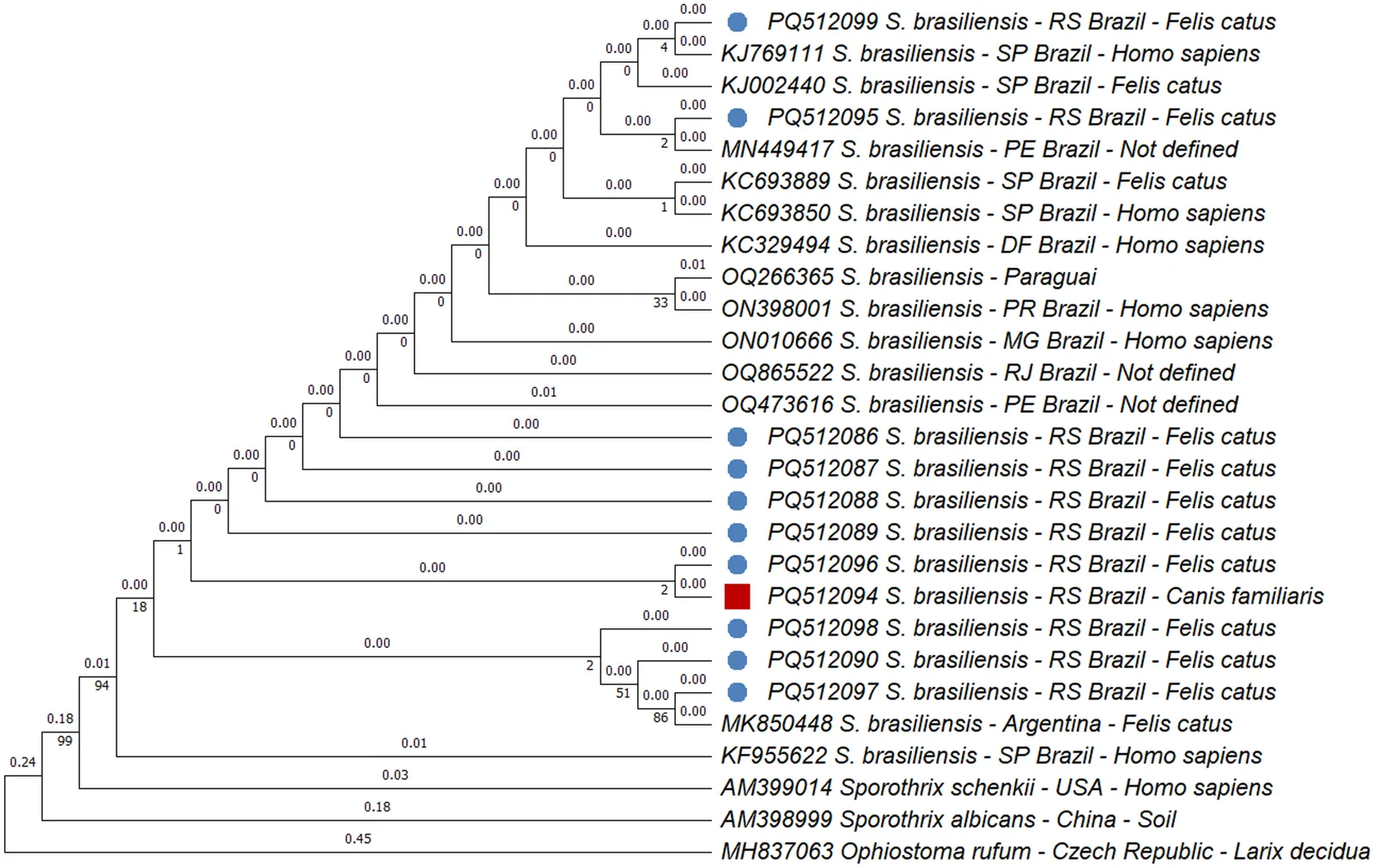
Maximum-likelihood phylogeny inferred from partial *calmodulin* (CAL) gene sequences of *Sporothrix* isolates from southern Brazil and reference strains. The tree was constructed using the Tamura 3-parameter model (T92) with 1,000 bootstrap replicates in MEGA 11 [19]. Blue circles represent feline isolates sequenced in this study, and red squares denote canine isolates sequenced in this study. *Candida albicans* and *Ophiostoma rufum* were used as outgroups. Bootstrap values ≥ 60% are shown at the nodes. Feline and canine isolates from Rio Grande do Sul formed a well-supported subclade within the *S. brasiliensis* lineage, which was closely related to isolates from Paraguay and Argentina, supporting evidence of cross-border dissemination in the Southern Cone. The scale bar indicates the number of nucleotide substitutions per site

Fourteen representative *CAL* sequences (~ 800 bp) were included in the Maximum Likelihood (ML) reconstruction under the Tamura 3-parameter (T92) model [18]. The resulting topology delineated a robust *S. brasiliensis* monophyletic clade (bootstrap support, BS ≥ 84%) clearly separated from *S. schenckii* and *S. albicans*. Within *S. brasiliensis*, moderate bootstrap values (BS = 60–84%) revealed limited intraspecific structure. A compact southern subclade comprising isolates from Rio Grande do Sul (predominantly feline, PQ512086–PQ512097) exhibited minimal nucleotide divergence (BS = 75–84). Sequences from São Paulo, Pernambuco, Minas Gerais, Paraná, and the Federal District formed a cohesive southeastern cluster (BS = 64–81). Two sequences from Paraguay (OQ266365) and Argentina (MK850448) nested within the *S. brasiliensis* clade proximal to southern Brazilian isolates, consistent with the cross-border dissemination in the Southern Cone. Intermixed human and feline sequences indicated the absence of host segregation, supporting sustained zoonotic transmission cycles mediated by domestic cats in this area. Overall, the *CAL*-based phylogeny confirmed *S. brasiliensis* as a genetically cohesive but regionally structured lineage (Fig. 3).

## Discussion

The predominance of *Sporothrix brasiliensis* in southern Brazil reinforces the ongoing territorial expansion of this species as the dominant zoonotic agent of sporotrichosis in the country [23]. The residual detection of *S. schenckii* suggests that ecological displacement is not yet complete and that ancestral *Sporothrix* lineages may persist in environmentally protected microhabitats within the Pampa biome [1, 24]. Such coexistence reflects ecological resilience rather than competitive exclusion, consistent with the adaptive capacity of *S. brasiliensis* to occupy urban and peri-urban niches through efficient cat-to-cat transmission [5]. In contrast, *S. schenckii* likely persists at low levels in environmental and alternative hosts.

Behavioral and management variables appear to be central to maintaining these dynamics. The predominance of infection in male cats (67%) and dogs (75%) mirrors previous evidence linking territorial behavior and aggressive interactions to the risk of inoculation [2, 6]. The very high proportion of semi-domiciled animals (93%), most of which had unrestricted outdoor access, further supports a model of sustained exposure to infected animals and short-range transmission networks [2].These behavioral patterns typify the urban endemic form of sporotrichosis, in which free-roaming males act as local amplifiers of the propagation of *Sporothrix*. This interpretation is consistent with recent modeling data from southern Brazil, where the basic reproduction number (R₀) for feline sporotrichosis was estimated to be 1.45 in Pelotas [25], confirming that transmission remains self-sustaining in the absence of coordinated control strategies in the region. Integrating behavioral ecology and housing management into control strategies remains essential [23], with confinement, neutering, and community education representing feasible interventions complementary to molecular and clinical surveillance.

The inclusion of 24 canine isolates expands the molecular–epidemiological framework of sporotrichosis in southern Brazil. Consistent with previous studies from southwest and southern Brazil [26, 27], *Sporothrix brasiliensis* was the only species detected, confirming the persistence of a unified domestic transmission network rather than host-specific cycles. The absence of phylogenetic segregation between feline and canine isolates indicates a shared epidemic pool in which dogs act as incidental yet epidemiologically informative hosts. These findings reinforce the importance of integrating canine sporotrichosis into One Health molecular surveillance systems in endemic regions.

Anatomical patterns also support this behavioral model of aggression. The lesions concentrated on the face, muzzle, and limbs, which are common sites of traumatic inoculation during fights, confirm that transmission in southern Brazil remains behaviorally mediated and ecologically stable [2, 6, 26, 28]. Spatially, the concentration of cases in Pelotas parallels observations from southeastern Brazil, where dense feline populations and unregulated movement sustain the endemic circulation of *Sporothrix* spp. [2, 5, 23]. Together, these findings delineate an endemic focus that persists under temperate conditions, which were previously considered marginal to the national epidemic.

Structural constraints inevitably limit the generalizability of these findings. Because sampling relied on passive laboratory submissions, isolates from peripheral municipalities were likely underrepresented, leading to a potential underestimation of the statewide incidence [7, 9]. In addition, feline sporotrichosis is frequently diagnosed by cytological examination, a rapid and highly sensitive method that often supports the initiation of treatment without the need for fungal cultures. In these situations, culture and molecular identification may not be performed, particularly in areas with limited access to specialized laboratories. Consequently, studies based exclusively on culture-confirmed isolates may underestimate the true burden and geographic distribution of sporotrichosis in the region [29]. Diagnostic access was further reduced during the COVID-19 pandemic (2019–2021) [29], and the proximity of another diagnostic facility in Rio Grande may have contributed to the uneven geographic distribution of the cases. Nevertheless, these limitations highlight a critical gap in regional surveillance capacity and underscore the need for a coordinated network linking veterinary and human laboratories to strengthen epidemiological surveillance and facilitate early detection.

Phylogenetic reconstruction confirmed that *S. brasiliensis* is a genetically cohesive, regionally structured lineage. The compact clustering of the isolates from Rio Grande do Sul suggests restricted gene flow and dominance of local transmission chains, which are features typical of expanding zoonotic lineages maintained by domestic reservoirs. This pattern is consistent with previous observations of low haplotypic diversity in endemic areas, supporting the notion that *S. brasiliensis* epidemics are driven by intense clonal propagation rather than by ongoing environmental recombination [1, 16].

The close phylogenetic affinity between southern Brazilian, Argentine, and Paraguayan isolates reinforces the hypothesis of a Southern Cone transmission corridor, where ecological continuity and companion animal mobility facilitate cross-border pathogen flow. Rather than stochastic dispersion, this genetic proximity likely reflects historical or contemporary seeding events mediated by human-associated movement and regional trade. The absence of pronounced divergence among isolates also supports the scenario of recent geographic expansion, consistent with the short evolutionary timescale inferred for *S. brasiliensis* epidemics [14, 23].

Although human isolates were not included, the proximity of animal-derived *S. brasiliensis* sequences to South American human reference strains indicates shared epidemiological relationships. This coherence corroborates previous evidence of genetically homogeneous populations circulating between animals and humans in endemic settings [2, 16, 23], emphasizing the utility of veterinary molecular surveillance as an early indicator of zoonotic risk in the One Health framework. Despite the reliance on CAL sequencing and passive submissions, the present study provides a consistent molecular–epidemiological signal for southern Brazil.

The predominance of *S. brasiliensis*, its phylogenetic clustering with neighboring South American isolates, and the behavioral profile of semi-domiciled cats collectively depict a stable zoonotic transmission cycle in the southernmost region of the continent. Within these boundaries, the CAL locus remains a robust and reproducible target for species identification and detection of lineage cohesion, validating its application as a first-line tool for molecular surveillance in veterinary and public health laboratories. Continued multicenter sequencing efforts, coupled with standardized clinical metadata, mandatory notification, and routine fungal culture and species identification, are essential to delineate the geographic limits of *S. brasiliensis* circulation, reduce reporting bias, and monitor its ecological persistence across the Southern Cone.

## Data Availability

Data supporting the conclusions of this study can be obtained from the corresponding author upon reasonable request.
